# Evaluating Novel Targets of Ischemia Reperfusion Injury in Pig Models

**DOI:** 10.3390/ijms20194749

**Published:** 2019-09-25

**Authors:** Andrea Baehr, Nikolai Klymiuk, Christian Kupatt

**Affiliations:** 1 Klinikum Rechts der Isar, Internal Medicine I, Technical University of Munich, 81675 Munich, Germany; n.klymiuk@tum.de (N.K.); christian.kupatt@tum.de (C.K.); 2 German Centre for Cardiovascular Research, Munich Heart Alliance, 80802 Munich, Germany

**Keywords:** I/R injury, ischemia reperfusion, pig model, preclinical research, cardiovascular animal model

## Abstract

Coronary heart diseases are of high relevance for health care systems in developed countries regarding patient numbers and costs. Disappointingly, the enormous effort put into the development of innovative therapies and the high numbers of clinical studies conducted are counteracted by the low numbers of therapies that become clinically effective. Evidently, pre-clinical research in its present form does not appear informative of the performance of treatments in the clinic and, even more relevant, it appears that there is hardly any consent about how to improve the predictive capacity of pre-clinical experiments. According to the steadily increasing relevance that pig models have gained in biomedical research in the recent past, we anticipate that research in pigs can be highly predictive for ischemia-reperfusion injury (IRI) therapies as well. Thus, we here describe the significance of pig models in IRI, give an overview about recent developments in evaluating such models by clinically relevant methods and present the latest insight into therapies applied to pigs under IRI.

## 1. Introduction

### 1.1. Mechanistic Principles

Coronary heart disease and consequent myocardial infarction (MI) play a prominent role as health risks, causing heart failure and premature death, particularly in Western societies. Evidently, tremendous research ambitions have been under way during recent decades to alleviate these problems. Despite all progress made in reperfusion therapies, a substantial proportion of heart failure patients (about 50%) have ischemic heart disease, most often aggravated by acute ischemic events. There is consent that reperfusion of vessels after acute ischemia may aggravate ischemic tissue injury [1,2]. Although the underlying mechanisms are complex, the most essential players have been known for more than a decade [3,4]: reactive oxygen species (ROS) [5], imbalance of Ca^2+^ homeostasis [6], mitochondrial damage [7] and, consequently, cell death [8]. Specifically, the reperfusion injury salvage kinase (RISK) [9] and survival activating factor enhancement (SAFE) [10] pathways have been explored in more detail as ever more interactions in ROS production became clear [11]. Additional aspects have been identified in the imminent role of mitochondrial damage [12,13] and downstream actors of modified signaling cascades such as the role of transcription factors in altered transcription [14] and the resulting changes in mRNA and miRNA levels [15] have been explored. Some evidence has also been gained that previously unknown players are involved in reperfusion injury: first, the Ca^2+^ imbalance [16] as well as the insulating effect of myofibroblasts and cardiac fibrosis [17] have been postulated as a causative for reperfusion arrhythmias. Second, autophagy [18,19] as well as the innate [20,21] and adaptive [22] immune system appeared major downstream actors of reperfusion injury. It is of note, that any of these effects play an ambiguous role, as they might act in both, a protective as well as in a detrimental manner. Finally, the very downstream event of cell death is not any more imputed to apoptosis [23], but rather to necroptosis, a programmed form of necrosis that appears to play an important role [24].

**Figure 1:**
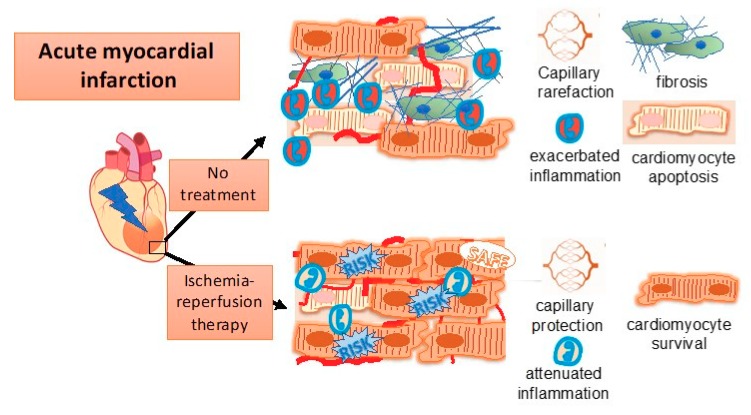
Key players in ischemia reperfusion injury are inflammation, endothelial dysfunction and cardiomyocyte damage.

The complex interplay of so many distinct processes in reperfusion injury (Figure 1) evidently hampers the development of sufficient treatments for myocardial infarction. Almost any axis has been targeted for improving I/R injury outcome, but the translation into sufficient clinical application has mostly failed. Multiple approaches using specific pharmacological compounds (reviewed in [25,26]) as well as numerous cellular transplantation attempts were made (reviewed in [27]). However, the somewhat conflicting outcome led to certain doubts regarding cardioprotective therapies in general [28]. Surprisingly, and in contrast to other holistic approaches of ischemic pre- or post-conditioning, remote ischemic conditioning during the occlusion period but before reperfusion (remote ischemic perconditioning), showed consistent improvement in numerous animals’ studies [29] and in a number of clinical trials [26,30,31]. Still, the degree of success leaves room for improvement and, thus, there is ongoing ambition to tackle reperfusion injury. However, evaluation of potential targets for improvement of ischemia/reperfusion injury can be challenging. Preclinical large animal models seem a complementary prequisite in the translational process towards clinical application, since rodent models for reperfusion injury not always predict therapeutic efficacy. 

### 1.2. Evaluating Ischemia-Reperfusion Injury in the Pig

Porcine animal models of myocardial ischemia that allow for the investigation of reperfusion injury have been established for almost three decades [32]. This preference may be explained by two main factors: first, pigs and their hearts come in a size that allows for use of patient-devised catheterization. Second, anatomical and physiological characteristics of the cardiovascular system, e.g., the lack of collaterals or the composition, the heart rate at rest and during exercise, and the post ischemic immune response, are closer to the human counterpart than any other species, except non-human primates. Reproducibility of infarct size normalized to area at risk, e.g., assessed by tetrazolium chloride (TTC) staining [33], and wealth of tissue for post-mortem investigation are further advantages of pig heart experiments. The high mortality of pigs after acute ischemia due to a high ventricular arrhythmogenic vulnerability has been a constant problem, which was tamed by the occlusion of the left anterior descending artery (LAD) distal to the first diagonal branch [34]. Finally, prevalent ethical considerations in society allow for the pig’s use in preclinical research, as opposed to dogs, cats and primates. First experiments on myocardial infarctions have been conducted in the 1960s and 1970s [35,36] and have been steadily improved and adapted since. Precise protocols are now available for enabling the transfer of models between the labs and for promoting better reference between them [33,37]. Equally a driving force are initiatives to align instrumentation and evaluation techniques with clinical standards, including modeling of cardiovascular risk factors such as diabetes [38] and hypercholesterinemia [39].

Significant changes in the field have occurred since the initial histological, structural and immunohistological evaluation of cardiovascular events in the pig [40]. They have been combined with clinic-grade imaging tools, e.g., magnetic resonance imaging (MRI): in a longitudinal study on reperfusion injury, cardiovascular magnetic resonance (CMR) revealed the 2-amplitude course of T2 relaxation in ischemic tissue within the first seven days post reperfusion [41]; in an acute setting, CMR was used to monitor ischemia-induced scar formation [42]; Gadolinium (Gd)-enhanced MRI was used to examine the post-conditional application of cardiosphere-derived cells (CDC) [43]; magnetic resonance imaging was used to determine T1 and T2 mapping in combination with Gd-contrast enhancement, phase-sensitive inversion recovery (LGE), cine-balanced steady-state free precession (bSSFP) and cine displacement encoding with stimulated echoes (DENSE) to analyze ischemia reperfusion injuries [44]. Compressed sensing has been used to further improve 3D multicontrast late enhancement for better identification of low spatiotemporal MR signals [45]. Recently, correlation of CMR-based elastography to mechanical testing of myocardial stiffness has been demonstrated also in the pig [46,47].

Apart from classical approaches for assessing immediate heart function, there is ambition in identifying reliable prognostic biomarkers for post ischemic patient cohorts. As an example, the volume of neutrophils appeared to be increased in post-MI patients as well as in MI pig models [48]. Upon systematic post-experimental asservation, the pig evidently facilitates insight at a cellular or molecular level. Preparation of cardiac tissue for 2D or 3D examination of heart anatomy has been described in a standardized way [49]. Preparation of tissue for confocal microscopy has been documented in an automated procedure [50]. Even 3D spatiotemporal dynamics has been explored in the pig heart by ultrasound-based strain imaging [51]. Evidently, and although annotation of the porcine genome is still significantly behind the status of human (or mouse), innovative next generation tools suggest the implementation of “-omics”, holistic genomic, transcriptomic or proteomic tools [52], with a particular emphasis not only on protein abundance, but also on their activation status [53]. Finally, in the age of artificial intelligence, the mathematical modelling and cumulative interpretation of raw data from different studies is gaining traction [54]. Protocols and endpoints have been established which allow correlation of preclinical outcome with future clinical examinations [55,56].

## 2. Promising Targets in I/R Injury

Targets for encountering I/R injury range from adaptations to hemodynamic parameters such as ischemic pre- and post-conditioning, microvascular protection [57], post ischemic inflammation [58], physical interventions (oxygen saturation, pressure or temperature, mechanical interventions) to pharmacological treatment, cell and exosome therapies [59] as well as bio-therapeutics that target specific regulatory cascades. Finally, also the combination of two or more of these approaches have been investigated in preclinical pig models.

### 2.1. Conditioning: Role of the RISK, SAFE and NO Pathways

The finding that manipulating or conditioning the immediate reperfusion process is feasible [1], has triggered numerous studies for improving the outcome of MI. The I/R injury treatment by remote ischemic perconditioning has been found to protect the porcine heart [60,61], but has only recently failed to demonstrate reduction of cardiac death or hospitalization for heart failure at 12 months in the Condi2-ERIC-PPCI trial [60]. Combining ischemic post-conditioning with administration of liraglutide, there is a tendency towards smaller infarcts, albeit non-significant [62]. In neonatal pigs, pre-conditioning appeared useful only in combination with continuous application of glucose and insulin [63]. As stated before, the RISK pathway, i.e. activation of PI3K-AKT- and MEK1-ERK1/2 [9], and the SAFE pathway, requiring activation of Stat3 [64], are potentially involved in reducing the impact of ischemia and reperfusion on the tissue [65]. Preconditioning with antagonists of the RISK and SAFE pathways revealed that only the latter is essential for the protective effect [65]. A study of remote ischemic pre- and perconditioning in a pig model of MI demonstrated activation of the PI3K-AKT pathway only by preconditioning the animal prior to MI but not by applying remote ischemic perconditioning during MI [66]. A transcriptome profile of pre-conditioned pig hearts supported the assumption that the activation status of STAT3 is indispensable in the stimulation of the SAFE pathway [67,68], as well as in remote perconditioning of ischemic pig hearts [69], but a specific stimulation of this pathway has not been tested yet in this species.

Trials for reducing infarct size have also included the application of bloodless reperfusion solutions containing oxygen carriers with and without additives such as glucose. However, no beneficial effect of these procedures could be found so far [70], although the delay of re-exposure of the myocardium to inflammation conveying blood components is attractive. High arterial oxygen reperfusion might, on the other hand, also cause excessive oxidative stress und as such aggravate I/R injury. Indeed, Abdel-Rahman et al. [71] could show that hypoxic reoxygenation in the early phase of reperfusion protects post ischemic myocardial function.

Inhibition of reperfusion induced thrombin has shown a beneficial effect on cardiac function after myocardial ischemia [72]. Carbon monoxide application can improve the energy status of post ischemic porcine hearts by increasing adenosine triphosphate (ATP) production. This leads to a decrease in edema formation and apoptosis, thus acting as a cardioprotective agent [73]. Similarly, preconditioning by nitric oxide (NO) application has improved capillary reperfusion by modifying myocardial oxygen consumption during reperfusion [74]. A longer lasting NO increasing intervention was performed by retroinfusion of the liposome-encoded active form of endothelial nitric oxide synthase (eNOS), which sufficed to decrease neutrophil invasion and infarct size [75]. In 2007, Eiferman et al. [76] could already show that systemic glucose-insulin-potassium infusion can improve myocardial recovery during reperfusion. This finding is in contrast to a previously mentioned study where glucose-insulin application had no significant effect on cardioprotection [63]. Importantly, however, the latter study focused on neonatal pigs in which the heart relies almost exclusively on carbohydrate metabolism. However, a metaanalysis of Zhao et al. of 23864 patients detected no benefit of glucose-insulin-potassium infusion in 11 randomized controlled trials [77].

### 2.2. Pharmacological Intervention

Pharmacological pre- and postconditioning has been investigated in regular intervals over the past decades. At times, different intervention targets came into fashion and fell out of vogue again. Na^+^/H^+^ exchange inhibitors seem to be a representative example. They appeared protective in I/R injury by preventing Na^+^ overload [78,79,80]. In combination with controlled reperfusion using leukocyte-depleted blood, Fedalen et al. [81] could confirm the protective effect of Na^+^/H^+^ exchange inhibitors. In own experiments, the combination of Cariporide and the antioxidant glutathione (GSH), but not the singular agents, attenuated porcine ischemia-reperfusion injury [82]. Consistently, no clear-cut benefit could be shown for Cariporide in the GUARDIAN trial of acute coronary syndrome patients [83].

Only recently, repurposing of pharmacological agents gained attraction, which is particularly the case for antihypertensive treatments. Among them, pre-treatment of a porcine MI model with eprosartan, showed improvement in regional cardiac function [84]. Similar improvement on hemodynamic parameters was obtained in ventricular fibrillation models upon treatment with nicorandil [85] and enalapril [86]. The application of diazoxide, a potassium channel opener of the mitochondria, also resulted in reduced MI damage [87]. It is, however, not known whether the drug acts indirectly by inducing vasodilation via relaxation of smooth muscle cells, or by altering osmolality in cardiomyocytes directly.

Further approaches targeting mitochondrial dysfunction were also mainly based on established drugs: TRO40303, a stabilizer of mitochondrial membrane permeability showed only very mild protection in a pig model of MI [88], and did not prove protective in a clinical trial of ST-elevation myocardial infarction (STEMI) patients [89]. Cyclosporine, preventing opening of the mitochondrial transition pore via cyclophiline [7], seemed to reduce infarct size (hyperenhancement on MRI) in a small patient cohort with acute MI [90], but failed to replicate the result in the larger Circus trial [91]. Ironically, in a previous porcine study, cyclosporine did not provide cardioprotection either [92]. In the preclinical arena, though, another stabilizer of the mitochondrial membrane, TVP1022, a modified version of the anti-Parkinson-drug rasagiline, prevented scar formation and improved cardiac function in MI [93]. No patient data are available as of yet.

On a more global level, the manipulation of the master regulator transcription factor Egr-1 that influences the key players of I/R injury, inflammation and apoptosis, leads to improved cardiac function after I/R [94]. Ivabradine, an inhibitor of cAMP-gated I*_f_* channels, has been investigated intensively in the past and revealed multiple cardioprotective effects [95], but did not show superiority to standard treatment in larger patient cohorts with myocardial infarction [96]. A similar fate was experienced by metformin [97,98] and carperitide [99]. In pigs, rapamycin, a mitosis blocker widely used as immunosuppressant and anti-restenosis drug on stents, surprisingly decreased cardiac function and induced myocardial necrosis [100]. Another multiple effective compound, however, deltorphin, an agonist of the delta opioid receptor, very recently prevented arrhythmia upon reperfusion in a porcine working heart model [101].

### 2.3. Biomimetics

A further trend in pharmacological conditioning is the increase in using biomimetic compounds, This is intriguingly illustrated by the case of anti-inflammatory or anti-oxidative approaches for MI in the pig [102,103,104,105], but has given way to biomimetics in the recent past, e.g., the unsuccessful clinical trial with glucocorticoids [106], which in preclinical rat, cat, rabbit and dog models has shown less devastating results (for review see [107]). Post ischemic sterile inflammation, with its orchestrated upregulation of cytokines and chemokines attracting neutrophils and monocytes/macrophages, is an often targeted process in ischemia-reperfusion treatment (for review see [58]). Although inhibition of endothelial activation with an anti-CD18 antibody (IB4) combined with NF Kappa B decoy oligonucleotides reduced early ischemia reperfusion injury in pigs [108], no singular agent was clinically successful to date, most likely due to the janus-faced nature of inflammatory cells, providing damage and repair at the same time. Novel approaches, such as administration of OPN-305, an anti-inflammatory clinical grade humanized anti-TLR2 antibody, led to improved cardiac function, albeit only at relatively high concentrations [104]. Moreover, application of the NLRP3-inflammasome inhibitor MCC950, in effect preventing formation of interleukin 1β, a pro-inflammatory cytokine initiating and maintaining post ischemic sterile inflammation, reduced infarct size and improved myocardial function after 75 min of LAD occlusion in pigs [109]. Using a gene therapeutic approach, cardioprotection by adeno-associated virus (AAV)-based hemoxygenase 1 (HO-1) overexpression decreased infarct size and post ischemic loss of function, apparently by reducing post ischemic neutrophil influx in pig hearts to a similar extent as ubiquitous HO-1 cardioprotection [110].

A cardiomyocyte-targeted approach such as AAV-mediated overexpression of myocardin related transcription factor A (MRTF-A), which induces expression of myocytic as well as angiogenic genes, improves ischemic myocardial tissue in a pig model of hibernating myocardium [111] and acute I/R. Interestingly, MRTF-A has been essential for maintenance of cardiomyocyte differentiation [112], similar to an upstream peptide of the MRTF-A/SRF pathway, thymosin beta 4 (TB4), which promotes differentiation towards cardiomyocytes [113]. Consistently, administration of either recombinant TB4 [114], or TB4 encoding plasmid or AAV-delivered TB4 [39] all proved cardioprotective. Moreover, AAV gene transfer of PR39, a pro-angiogenic protein which induces the transcription factor HIF1alpha, in turn attenuates MI in pigs [115]. In addition, a silencing oligonucleotide against the promoter region of EGR1 was used in a pig model of MI [94], whereas the application of miRNAs revealed that also post-transcriptional regulation at RNA level might be valuable in preventing reperfusion damage [116]. Finally, an inhibitor of microRNA92a (LNA-92a) exerted pleiotropic effects on cardiomyocyte survival, attenuation of neutrophil influx as well as capillary preservation [117].

Another essential field of intervention for I/R injury appears to be the immediate stabilization of damaged tissue. Mitsugumin53 (MG53), a proposed stabilizer of membranes, was used to prevent I/R injury [118,119] and a first-in-pig study had added evidence [120], but since then no further pre-clinical examination has been presented. Another approach focused on a steroid component of membranes, cholesteryl esters: again, stabilization of membranes improved IRI outcome [121]. A similar attempt was followed with rotigaptide, a hexapeptide that enhances electrical coupling of cardiomyocytes by modulating connexin activity [122]. Very differently, tissue damage was successfully prevented by apheresis of C-reactive protein from the circulation [123].

### 2.4. Cellular Treatments

Longterm cell supplementation for lost or dysfunctional parenchymal or vascular cells is not an easy task in the heart. Despite the mixed results of clinical study metaanalyses [125,126], intramyocardial injection of 1 × 10^7^ bone marrow derived cells improved LV diameters and EF at 3 months after MI [127], similar to bone marrow mesenchymal stem cell transplantation induced glucose transporter genes as well as mTOR signaling in 4-week-old porcine infarct areas [128]. Beyond bone-marrow cells, cardiac-derived cells (CDC) were applied either by intracoronary infusion [129,130,131] or by intra-myocardial injection [132] in pig hearts, preventing loss of cardiac function post MI and improving myocardial perfusion [131]. The Scipio trial, a clinical phase I study, showed encouraging results [133], the Caduceus trial reported an increase in viable myocardium in the infarct zone of 17 MI patients [134].

Further advancing the field and simplifying the approach are exosomes, as small (30–100 nm) vesicles derived from luminal membranes of multivesicular bodies that are capable of carrying protein, mRNA or microRNA cargo [135]. CDC-derived exosomes, applied either by intracoronary infusion or by intramyocardial injection, sufficed to prevent adverse remodeling in pig hearts [136,137]. The latter study found that fibroblast-derived exosomes did not result in the same benefit unless they were spiked with miR-181b miRNA. Other factors isolated from plasma-derived exosomes include heat shock protein 70 (Hsp70) and pregnancy-associated plasma protein-A (PAPP-A) [59].

For providing a supportive environment of endogenous or exogenously applied cells, extracellular matrix hydrogel was injected into the myocardium two weeks post MI and resulted in an improved endocardial muscle content with reduced fibrotic tissue [138]. This material itself did not provide improvement of adverse cardiac remodeling in the heart in patients, but is potentially useful as a therapeutic niche for further cardiac cell seeding, given a favorable safety profile [139]. Furthermore, the extracellular matrix protein agrin contains potential for cardiac regeneration in mice [140], and is currently assessed for its potential to attenuate ischemia-reperfusion injury in pigs (E. Tzahor, personal communication).

Closing the gap to mature, functional cardiomyocytes, human embryonic stem cell derived cardiomyocytes were engrafting in the infarcted apex of pig hearts, mounting a trend towards more wall-thickening at 4 weeks post-MI. On the other hand, a substantial number of, at times fatal, ventricular tachycardias was detected in between [141]. Engineered heart tissue derived from human induced pluripotent stem cells (iPSCs), which beneficially affected cardiac remodeling [142] and did not spark ventricular arrhythmias in a guinea pig cryo-injury model [143], are currently tested in porcine models.

Conceptually, many cell-therapeutic approaches foresee a xenogeneic situation and complex control of immune rejection in pigs, triggering the use of immunocompromised pigs, e.g., by identification of naturally occurring mutations or by genetic engineering [144]. Most of these immunodeficient models suffer from defects in ILR2G, RAG1, RAG2 or Artemis, requiring suitable housing under specific pathogen free (SPF) conditions [145]. We have recently developed an alternative pig model that proved immune-deficient due to the transgenic expression of the T-cell activation inhibitor LEA29Y [146], which upon maintenance in an SPF facility [147] has shown effective natural reproduction (unpublished data). Such animals might be appropriate for testing cellular transplants in I/R injury.

## 3. Summary & Outlook

The intensive research that has been done on pre-clinical evaluation of treatment options for myocardial infarction in pigs in the recent past illustrates the relevance of this species in bridging the gap from basic research to clinical application. Key areas of development are (1) modifiers of post ischemic inflammation (inflammasome inhibitors, cytokine/chemokine inhibitors), (2) stabilizers of mitochondrial and metabolic functions of cardiomyocytes and (3) protective agents for microcirculatory structure and function. Several principles such as enhancing the MRTF-A/SRF pathway, affect more than one of these processes [111]. Still, many studies described here are not fully comparable in detail, precluding a ranking of I/R injury targets in a defined manner. As shown in Table 1, a variety of pharmacological agents have been assessed in pigs, which in most cases matched available clinical data. Nevertheless, preclinical large animal studies do not always accurately predict clinical studies, e.g., remote ischemic conditioning and anti-inflammatory therapies. A heterogeneity issue in larger clinical cohorts with regard to risk factors, uncontrolled ischemia length in patients, heterogeneity of patient age, training state, pharmacotherapies and co-morbidities is notoriously hard to model [148,149], although pigs of different ages, with one or more cardiovascular risk factors (diabetes, hyperlipidemia, hypertension) [38,150,151], are available. However, application of a standardized, good manufacturing practice (GMP) grade, biological agent is more difficult than in small animals. Moreover, the specificity of the agent may vary from species to species, e.g., in the field of non-coding RNAs or cell-based treatments for I/R injury.

In summary, the adaptation of novel imaging technologies and the availability of genetically modified pig models are cornerstones in advancing pig pre-clinical models for testing I/R injury treatments. It is safe to assume that application routes, dosages and timing of novel therapeutics can be elaborated in pig models which are closely resembling clinical scenarios. In addition, porcine studies may aid in elucidating mechanisms of action as well as efficacy of therapeutic agents, which are evolving from basic research or clinical studies as attractive in treating ischemia and reperfusion injury.

## Figures and Tables

**Table 1:** Pharmacological agents assessed in porcine/human studies of ischemia-reperfusion.

Agent	Outcome in Pigs	Outcome in Patients (Largest Study Cited)
cariporide (N^+^H^+^ exchanger inhibitor)	not significant [82]	neutral (Guardian) [83]
cyclosporine (mitochondrial permeability transition pore inhibitor, immunosuppressant)	not significant [92]	not significant (Circus) [91]
carperitide (synthetic ANP)	dP/dt better [99]	not defined
glucose-insulin infusion	earlier recovery after mild ischemia [76]	no benefit [77]
ivabradine (I*_f_* channel blocker)	significant	not significant
MCC950 (NLRP3 inflammasome inhibitor)	infarct size reduced, EF better [109]	not defined
metformin (oral antidiabetic drug)	not significant (LV function) prevents arrhythmias [98]	TIMI 53 (post hoc analysis) overall mortality reduced, no effect on CV mortality, MI, stroke [124]
mitsugumin53 (RISK pathway initiator, binds PI3K to CaV3)	significant	not defined
OPN-305 (anti-TLR2 antibody, IL6 inhibitor)	infarct size reduced FS better [104]	not defined
TRO40303 (mitochondrial membrane stabilizer)	not significant [88]	not significant (Mitocare) [89]
TVP1022 (rasagiline-derivate, anti-Parkinson)	infarct size reduction significant [93]	not defined
LNA-92a (inhibition of microRNA 92a)	infarct size reduced neutrophil influx decreased, LV function improved at 72h and 7d (significant) [117]	not conducted yet

LV = left ventricle, FS = fractional shortening, EF = ejection fraction, dP/dt = contraction velocity, CV = cardiovascular, h = hour, d = day.
